# Therapeutic Effects of Single and Combined Anti-Disseminated Intravascular Coagulation (DIC) Drugs in a Rat Venom-Induced Consumption Coagulopathy (VICC) Model Using Yamakagashi (*Rhabdophis tigrinus*) Venom

**DOI:** 10.3390/toxins18030151

**Published:** 2026-03-23

**Authors:** Akihiko Yamamoto, Takashi Ito, Toru Hifumi

**Affiliations:** 1 Management Department of Biosafety and Laboratory Animal, and Pathogen Bank, National Institute of Infectious Diseases, Tokyo 208-0011, Japan; 2 Department of Biomedical Laboratory Sciences, Faculty of Life Sciences Kumamoto University, Kumamoto 860-8556, Japan; tito@kumamoto-u.ac.jp; 3 School of Medicine, Kyorin University, Tokyo 181-0004, Japan; hifumitoru@gmail.com

**Keywords:** yamakagashi (*Rhabdophis tigrinus*) venom, rat venom-induced consumption coagulopathy (VICC) model, recombinant thrombomodulin alpha, tranexamic acids, antithrombin III, combined therapy

## Abstract

Yamakagashi (*Rhabdophis tigrinus*) is a widely distributed snake species in Japan. Yamakagashi causes venom-induced consumption coagulopathy (VICC) when the amount of infused venom is high, and bites can be fatal if antivenom treatment is delayed. However, yamakagashi antivenom is an unapproved treatment, and its storage capacity is limited, preventing its prompt administration. Therefore, we investigated the application of commercially available drugs, namely tranexamic acid and antithrombin III, in the treatment of VICC caused by yamakagashi venom in a rat model. Furthermore, we investigated the combination of each drug with recombinant thrombomodulin α. Administration of tranexamic acid or antithrombin III alone failed to extend rat survival or correct changes in blood coagulation markers, such as prothrombin time, fibrinogen concentrations, and D-dimer levels, in yamakagashi venom-treated rats. However, combined administration of recombinant thrombomodulin α and tranexamic acid extended rat survival and partially restored blood coagulation markers. Therefore, the combination of recombinant thrombomodulin α and tranexamic acid might represent a useful therapeutic regimen for yamakagashi venom exposure.

## 1. Introduction

Bites from snakes, including yamakagashi (*Rhabdophis tigrinus*), can cause venom-induced consumption coagulopathy (VICC) [1], a severe clinical condition characterized by marked, persistent, and widespread activation of circulating blood coagulation, resulting in the formation of microvascular thrombi. Patients experienced hypo- or afibrinogenemia [2,3]. Yamakagashi is a venomous snake belonging to the Colubridae family, and it is typified by short posterior fangs [4]. This snake inhabits East Asia, being commonly found in rice paddies in Japan, where it primarily consumes frogs [5]. As a rear-fanged snake, Yamakagashi bites are rarely severe, as their venom penetrates only human skin if the bite from their rearmost fangs is sustained for a certain period. However, several bites requiring medical treatment are reported annually [6]. Severe cases can be fatal without treatment, and deaths have been previously reported [7].

Regarding the chemical properties of yamakagashi venom, it has been reported that the high molecular weight portion of the venom contains a prothrombin activator. It has been reported that when yamakagashi venom acts, both the prothrombin time (PT) and the activated partial thromboplastin time in human plasma are shortened [6,8,9]. In previously reported studies, fractions of yamakagashi venom did not hydrolyze matrix metalloproteinase substrates such as collagen and laminin, and showed specific protease activity against human fibrinogen [10]. In addition to localized hemorrhage, systemic subcutaneous, pulmonary, and subendocardial hemorrhage has also been observed in mouse experiments. Microthrombi have been detected in alveolar capillaries and glomeruli following yamakagashi venom exposure [11]. Systemic hemorrhage, VICC, and acute renal failure have been recorded following bite injuries [12,13], and two fatal cases attributable to acute pulmonary edema and cerebral hemorrhage have been reported [14,15].

Currently, yamakagashi antivenom is used to treat poisoning associated with venom exposure, but its use is limited by its lack of regulatory approval. Yamakagashi antivenom is obtained from the plasma of horses immunized with the snake’s venom. Administration of these preparations, which contain foreign proteins, to humans carries the risk of side effects such as serum sickness and animal protein-induced anaphylaxis [16]. For VICC caused by such yamakagashi bites, there are medications available for DIC, which presents with symptoms like blood clotting disorders [17,18,19,20]. As DIC has diverse causes [1,2], these drugs have not been used to treat VICC caused by venomous snake bites, but they are readily available in clinical settings. In 1982, Dr. Esmon et al. discovered thrombomodulin α as a glycoprotein present in vascular endothelial cells. This molecule is a physiological anticoagulant that regulates blood coagulation in the body [21]. In the treatment of DIC, recombinant thrombomodulin α (rTM) has been reported to promote the activation of protein C by thrombin, and this activated protein C then uses the coagulation factor protein S to degrade factors Va and VIIIa, thereby suppressing blood coagulation by generating thrombin [22]. In a previous study, we demonstrated that rTM neutralizes the coagulation activity of yamakagashi venom in vitro and reported its potential therapeutic efficacy in vivo [23]. However, subsequent studies using increased doses did not demonstrate sufficient therapeutic efficacy in vivo.

Tranexamic acid (TXA), which is used to treat hyperfibrinolytic DIC [24,25], prevents plasmin from binding to fibrin, thereby suppressing overactivation of the fibrinolytic system. Meanwhile, antithrombin III (ATIII) inhibits overactivated blood coagulation and prevents thrombus formation in microvessels [26]. DIC results in frequent thrombus formation and organ damage, and coagulation factors, including ATIII, are consumed in large quantities. Therefore, ATIII supplementation is used to calm the hypercoagulable state, restore the balance between coagulation and fibrinolysis, and improve bleeding and thrombosis [2,3,4,5,6,7,8,9,10,11,12,13,14,15,16,17,18,19,20,21,22,23,24,25,26,27,28,29].

Given that yamakagashi antivenom is not readily available in clinical settings, we considered the application of more readily available DIC drugs. We investigated the potential efficacy of TXA and ATIII monotherapy, as well as their combined use with rTM, in the treatment of VICC caused by yamakagashi bites in a rat model.

## 2. Results

### 2.1. Life-Saving Effect of ATX and ATIII in the Rat DIC Model

**Figure 1:**
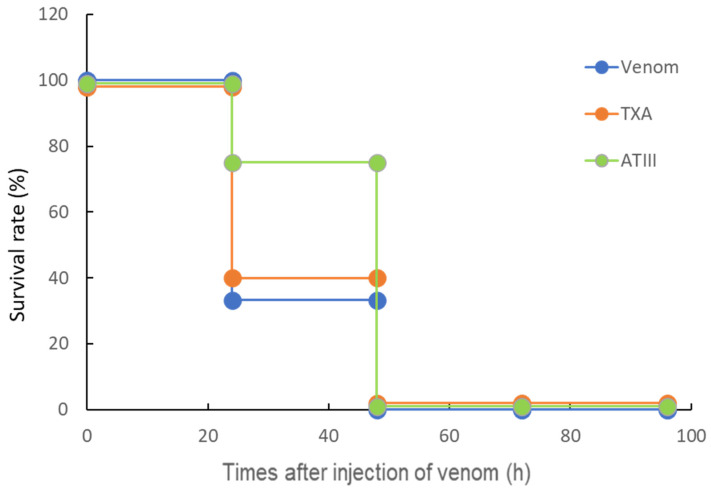
Effects of tranexamic acid and antithrombin III against lethal doses of yamakagashi venom. Blue circles indicate the duration of survival after intramuscular administration of 300 μg of venom. Orange indicates the duration of survival when tranexamic acid was administered 30 min after venom administration. Green indicates the duration of survival when antithrombin III was administered 30 min after venom administration. Each group comprised six rats. Survival data were analyzed using the Kaplan–Meier method.

We used a previously described rat VICC model to measure the in vivo activity of TXA and ATIII monotherapy [30]. Briefly, 30 min after venom administration, TXA (5 mg/rat) or ATIII (20 U/rat) was intravenously administered to the rats. As presented in Figure 1, administration of TXA (5 mg/rat) or ATIII (20 U/rat) alone did not improve rat survival.

### 2.2. Effects of TXA and ATIII on Blood Coagulation Markers in a Rat VICC Model Induced by Yamakagashi Venom

**Figure 2:**
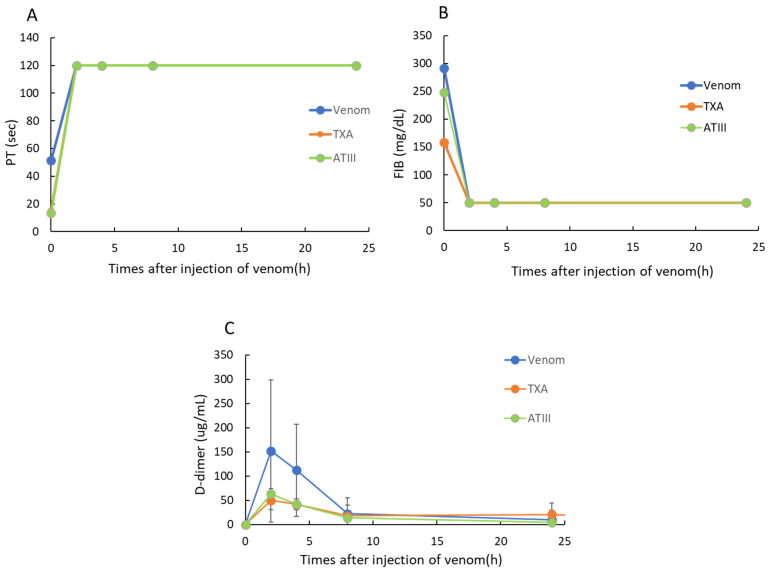
Time course of (**A**) PT, (**B**) FIB concentrations, and (**C**) D-dimer levels after administration of 300 μg of yamakagashi venom to rats. In each panel, blue circles indicate the time course after venom administration alone, orange circles indicate the change after TXA administration 30 min following venom exposure, and green circles indicate the change after ATIII administration 30 min following venom exposure. Each group included six rats. Data are presented as means and SDs.

We next investigated changes in blood coagulation markers (PT, FIB concentrations, and D-dimer levels) in the rat VICC model (Figure 2). Two hours after yamakagashi venom exposure, PT was delayed to 120 s (the limit of detection), FIB levels decreased to below the limit of detection, and D-dimer levels exhibited a transient increase that peaked at 2 h. Conversely, in the TXA- and ATIII-administered groups, PT and FIB levels were numerically lower after 2 h, but no significant differences were observed versus the venom-administered group. Meanwhile, D-dimer levels were suppressed by approximately 30% following TXA or ATIII administration.

### 2.3. Effects of rTM, ATIII or TXA Alone, and TXA or ATIII in Combination with rTM on Survival in a Rat Model of VICC Induced by Yamakagashi Venom

**Figure 3:**
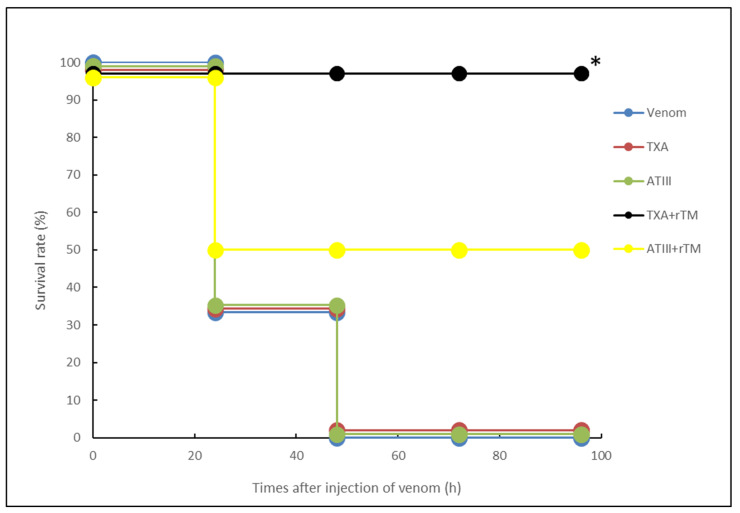
Combined effects of rTM plus TXA or ATIII against a lethal dose of yamakagashi venom. Blue circles indicate survival after intramuscular administration of 300 μg of venom. Black circles indicate survival after rTM and TXA treatment in venom-exposed rats. Yellow circles indicate survival after rTM and ATIII administration in venom-exposed rats. Orange circles indicate survival after TXA administration alone in venom-exposed rats. Green circles indicate survival after ATIII administration in venom-exposed rats. Survival data were analyzed using the Kaplan–Meier method. Each group consisted of six rats. * *p* < 0.05.

Next, we conducted an in vivo assay to examine the therapeutic efficacy of TXA and ATIII, either alone or in combination with rTM, in treating yamakagashi venom exposure. As presented in Figure 3, 50% of venom-exposed rats survived after treatment with the combination of rTM and ATIII, whereas all venom-exposed rats that received the combination of rTM and TXA survived for 96 h. Conversely, TXA (5 mg/rat) or ATIII (20 U/rat) alone did not improve rat survival.

### 2.4. Therapeutic Effects of rTM Plus TXA or ATIII on Blood Coagulation Markers in Rats with Yamakagashi Venom-Induced VICC

**Figure 4:**
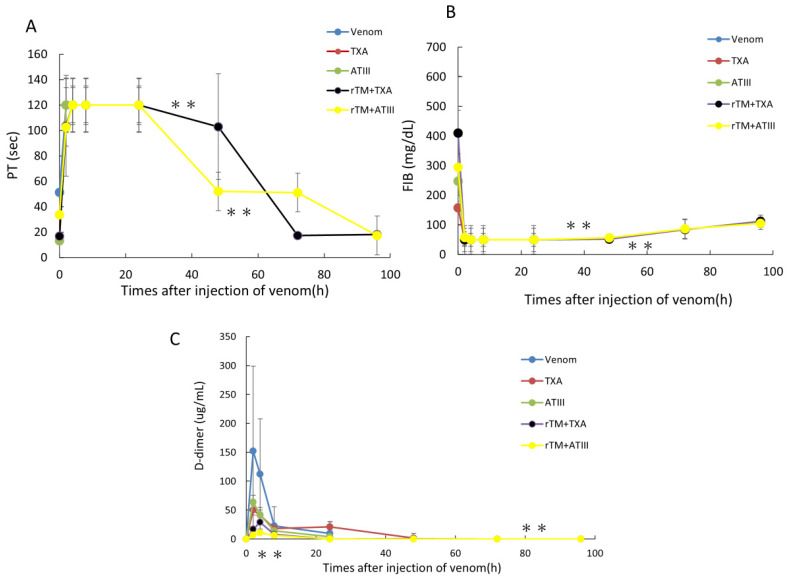
Time course of PT, FIB concentrations, and D-dimer levels after administration of 300 μg of yamakagashi venom to rats. (**A**) Time course of PT. (**B**) Time course of FIB concentrations. (**C**) Time course of D-dimer levels. Blue circles indicate changes in blood coagulation markers (PT, FIB concentration, and D-dimer levels) in rats intramuscularly administered 300 μg of venom alone. Black circles indicate changes in blood coagulation markers in venom-administered rats treated with rTM and TXA. Yellow circles indicate changes in blood coagulation markers in venom-administered rats treated with rTM and ATIII. Orange circles indicate changes in blood coagulation markers in venom-administered rats treated with TXA alone. Green circles indicate changes in blood coagulation markers in venom-administered rats treated with ATIII alone. Each group consisted of six rats. In the in vitro blood coagulation system comparison, the parallel line assay method was used. In addition, the binary placement method was used to compare blood coagulation markers. Data are presented as means and SDs. ** *p* = 0.01.

Based on the life-saving effects of rTM combined with TXA or ATIII in the rat VICC model, we examined changes in blood coagulation markers (PT, FIB concentration, and D-dimer levels). At 2 h after venom administration, PT was delayed to the measurement limit of 120 s, the FIB concentration was decreased to below the limit of detection, and D-dimer levels were transiently increased. No improvements in blood coagulation factors were observed at 2 h after venom administration in rats treated with TXA or ATIII alone. Conversely, in the combination treatment groups, PT levels recovered over time, FIB levels partially restored and D-dimer levels remained depressed (Figure 4).

## 3. Discussion

Based on our previous research into the potential use of rTM in treating yamakagashi bites [30], we initially investigated the efficacy of TXA and ATIII as single agents in our rat VICC model. As both drugs can be administered early to patients after Yamakagashi bites, they were administered 30 min after venom administration. TXA and ATIII are both commercially available anti-DIC drugs with different mechanisms of action. TXA binds strongly to the lysine-binding site, which is the fibrin affinity site of plasmin and its precursor plasminogen, which participate in fibrinolysis occurring after blood coagulation [31]. Through this binding, TXA prevents plasmin and plasminogen from binding to fibrin, thereby strongly inhibiting fibrin degradation by plasmin. Furthermore, the antifibrinolytic effect of TXA is further enhanced in the presence of plasma antiplasmins such as α2-macroglobulin [32]. Plasmin inhibits platelet aggregation and degrades coagulation factors, but it specifically induces fibrin degradation. Therefore, TXA stops bleeding by inhibiting fibrin degradation [33,34]. Meanwhile, ATIII has broad serine protease inhibitor activity, and it has an important role in controlling blood coagulation. In vitro, ATIII inhibits the activity of intrinsic coagulation factors, such as thrombin, factor Xa, factor IXa, factor XIa, factor XIIa, and plasma kallikrein, but physiologically, it exerts an inhibitory effect on the central coagulation reaction, including thrombin and factor Xa [35,36].

Our previous analysis has identified prothrombin activator, metalloproteinase, and cysteine-rich secretory protein (CRISP) as components of the venom of the yamakagashi, and some of their properties, such as the toxicity of the venom and the demonstration of prothrombin activator and metalloproteinase, have been studied [14,37]. Metalloproteases have been described as the molecules responsible for the blood clotting action of yamakagashi venom [10,37]. It is speculated that accelerated coagulation that occurs in rats administered with venom, resulting in the secondary phenomenon of deficiency of FIB coagulation factors [37]. This accelerated coagulation is followed by increased fibrinolysis, leading to bleeding tendency [6]. Increased fibrinolysis degrades fibrin produced by coagulation, resulting in an increase in D-dimer levels, as observed in the present study. Although TXA can inhibit fibrin degradation, this effect was not observed in our experiments using TXA alone. Furthermore, although ATIII exhibits serine protease inhibitory activity, it has been reported that the activity of metalloproteases in yamakagashi venom cannot be inhibited by serine protease inhibitors [10]. This suggests that ATIII alone cannot neutralize yamakagashi venom.

Next, we evaluated the therapeutic efficacy of combinations of commercially available anti-DIC drugs in our rat VICC model. Surviving rats exhibited significant reversal of PT prolongation and FIB levels, alongside significant suppression of the transient increase in D-dimer levels. Thrombomodulin α, a glycoprotein found in vascular endothelial cells, acts as a physiological anticoagulant and regulates blood clotting in the body [22,37,38,39]. rTM is a drug used to treat DIC caused by various factors, including infections, cancer, sepsis, and other diseases [40,41,42]. Our previous report on the potential of rTM for treating yamakagashi bites suggested that its therapeutic efficacy could be enhanced in combination with other anti-DIC drugs [23]. Although ATIII alone exhibited no therapeutic effect on the coagulation activity of snake venom, promising activity was observed when ATIII was used in combination with an antivenom that was similarly ineffective on its own [43,44]. Considering these reports, ATIII might function as a secondary complement factor, albeit with limited efficacy, even if it does not directly affect yamakagashi venom.

Although TXA did not exert therapeutic benefits alone, its combined use with rTM led to improvements in survival and blood coagulation markers in the rat VICC model. The mechanism by which rTM inhibits blood coagulation is as follows: First, it promotes the activation of protein C by thrombin. This activated protein C uses protein S as a coagulation factor and cleaves factors Va and VIIIa in the coagulation factor pathway to produce thrombin [22]. In rats administered yamakagashi venom, accelerated coagulation occurs, presumably secondary to a deficiency in FIB [37]. This accelerated coagulation is followed by increased fibrinolysis, resulting in bleeding [6]. Thus, combinations of drugs with different mechanisms of action can produce enhanced effects, as observed in this experiment [43,44]. In fact, there are reports TXA use in patients with venomous snakebites [45], and it is actually used clinically to suppress bleeding tendency following snakebites. Furthermore, the sequential use of rTM and TXA has been applied in the treatment of DIC [46,47], suggesting that the therapeutic effect observed in our rat VICC model in this experiment could reflect actual clinical efficacy.

One limitation of this study is the lack of knowledge regarding the mechanism of action of the combination of rTM and TXA in treating yamakagashi venom-induced DIC, although their individual mechanisms have been clarified. Therefore, future research considering the mechanism of action of the two drugs in combination could expand the possibility of using rTM and TXA in clinical practice.

## 4. Conclusions

The combined effects of rTM and TXA, and rTM and ATIII were investigated in rat VICC models induced by the venom of the yamakagashi snake. The results showed that the combined effect of rTM and ATIII extended the survival time of 50% of rats, and the combined administration of rTM and TXA extended the survival time of almost all rats. Furthermore, partial recovery of blood coagulation markers was confirmed. Therefore, this combination therapy may be useful in the treatment of yamakagashi bites.

## 5. Materials and Methods

### 5.1. Animal Preparation

Twelve-week-old male Sprague Dolly rats (SLC Japan Co., Ltd., Shizuoka Prefecture, Japan) were housed in individual cages in a temperature-controlled room on a 12-h light/12-h dark cycle. The rats were fed standard laboratory feed and given free access to water. Larger, heavier rats were used to facilitate cannulation of the femoral artery and vein. The 12-week-old male Sprague Dolly rats used in the experiment weighed 300–350 g. All surgical procedures and experimental steps performed on the rats were approved by the Animal Husbandry and Use Committee. Experimental techniques and rearing methods were also carried out in accordance with animal experiment guidelines (Approval number: 118065, Approval date: 13 December 2018).

Under general anesthesia with 4% isoflurane, a polyethylene catheter (PE-60) was inserted into the femoral artery of each rat to collect blood. Another catheter (PE-50) was inserted into the femoral vein to administer saline solution and drugs. After blood collection and drug administration, all catheters were filled with heparinized saline solution (100 U/mL) and placed subcutaneously. An opening was then made at the back of the neck to prevent the catheters from moving when the animals woke up. After the two catheters were in place, the rats were allowed to recover for 48 h before being used in the experiment. Blood collection and drug administration were performed under anesthesia with 4% isoflurane.

### 5.2. Snake Venom

Crude venom of yamakagashi was extracted from the Duvernois glands. A pair of Duvernois glands, located on either side of the jaw, were collected from 100 snakes of unknown sex and over 80 cm in length. The excised glands were finely chopped, and the venom components were extracted by adding an appropriate amount of distilled water. The supernatant of the extracted venom was freeze-dried after centrifugation. The venom powder was again dissolved in distilled water, centrifuged again to remove mucus that had been mixed in during venom extraction, and freeze-dried to obtain the crude venom [30]. The freeze-dried venom was stored in a refrigerator. The intravenous LD50 value of this venom was 0.265 mg/kg in mice [14].

### 5.3. Experimental Protocols

To evaluate the effects of TXA or ATIII alone, rats were randomly divided into three groups, and mortality and three plasma coagulation-related factors (PT, FIB, and D-dimer) were assessed. The model groups used were those previously reported to have been used to investigate the routes and doses of venom administration for the creation of a rat VICC model using yamakagashi venom [30]. 300 μg of yamakagashi venom was administered intramuscularly. In the TXA group, 5 mg of TXA was administered intravenously 30 min after venom administration. In the ATIII group, 20 U of ATIII was administered intravenously 30 min after venom exposure. The dose of each anti-DIC drug was calculated by converting the maximum clinical dose in humans to the weight-based equivalent in rats. Each group consisted of six rats. Blood samples were collected at 0, 2, 4, 8, 24, 48, 72, 96, and 120 h after the start of the experiment (1500 μL of blood was collected at each time point). The timing and volume of blood sample collection followed the methods used in the previously reported rat model, based on the changes in blood coagulation factors and the survival curves of rats [30]. After collection, the same volume of saline was administered via femoral vein. The blood samples were treated with sodium citrate for anticoagulation, and then immediately centrifuged at 3000× *g* for 10 min to separate the plasma.

To evaluate the combined effects of rTM with TXA or ATIII, rats were randomly divided into five groups, and mortality and plasma coagulation-related factors (PT, FIB, and D-dimer) were assessed. In the model group, 300 μg of yamakagashi snake venom was administered intramuscularly. In the TXA group, 5 mg of TXA was administered intravenously 30 min after venom administration. In the ATIII group, 20 U of ATIII was administered intravenously 30 min after venom administration. The dose of each anti-DIC drug was calculated by converting the maximum clinical dose in humans to the weight-based equivalent in rats. In the rTM + TXA group, 1 mg/kg of rTM was administered intravenously 10 min after venom administration, followed by 5 mg of TXA 30 min later. In the rTM + ATIII group, 1 mg/kg of rTM was administered intravenously 10 min after venom administration, followed by 20 U of ATIII 30 min later. Each group consisted of 6 rats. The timing of blood collection and plasma separation followed the timing used in the monotherapy experiment. Based on a previous study [23], rTM was administered intravenously at a dose of 1 mg/kg for 10 min after yamakagashi venom administration. ATIII was administered intravenously at 20 U/rat, calculated from the clinical dose, for 30 min after venom administration.

### 5.4. PT Measurement

An Individual plasma samples were placed in test tubes for measurement using a CA-101 automated analyzer (Sysmex Corporation, Kobe, Japan). In this coagulation detection method, a mixture of plasma and reagent is irradiated with red light (660 nm) to measure PT. When fibrin clots form due to blood coagulation, changes in turbidity are detected as changes in scattered light, and then the coagulation time (PT) is measured. The typical maximum detection time for PT is 120 s. The coefficient of variation (CV) of PT measurement is less than 2%. The CV is based on the change in activity (%) in 10 analyses of Dade Behring Ci-Trol Level 1 (control plasma) using the PT reagent.

### 5.5. FIB Measurement

For FIB measurement, individual plasma samples diluted 10-fold with Owren’s buffer were placed in the measurement tubes of the CA-101 automated analyzer. The analytical range for FIB concentration with this instrument is 50–450 mg/dL. The coefficient of variation (CV) for FIB measurement is less than 4%. CV was calculated based on the percentage change in activity when Dade Behring Ci-Trol Level 1 (control plasma) was analyzed 10 times with Dade Behring fibrinogen assay reagent.

### 5.6. D-Dimer Measurement

Plasma D-dimer concentrations from individual rats collected in the experiment were determined in the same manner as previously reported [23]. Specifically, they were measured using latex photoimmunoassay (LPIA)-NV7 with the LPIA ACE D-dimer II kit (LSI Medience, Tokyo, Japan). The measurement method was as follows: First, 4 μL of plasma was collected and dispensed into a reaction cuvette containing 144 μL of R-1 solution. This mixture was stabilized by heating at 37 °C for 2 min, after which 48 μL of R-2 suspension containing latex particles coated with anti-D-dimer antibody JIF-23 was dispensed. The increase in turbidity of this solution was evaluated during a reaction by heating at 37 °C for 7 min. The analytical range of D-dimer concentration using this method is 0.5–48 μg/mL. Samples exceeding this analytical range were diluted and re-measured. The intrarun coefficient of variation (CV) in this measurement was less than 10%. Preliminary tests in which rat plasma samples were treated with calcium ions and tissue plasminogen activator at various concentrations confirmed cross-reactivity between the antibody and rat D-dimer.

### 5.7. Statistical Analysis

This paper explains the statistical methods used. First, the uniformity of the data distribution in each experimental group was evaluated using the Bartlett test. Bidirectional analysis was used to compare data groups where uniformity was confirmed by this test. On the other hand, the Mann–Whitney U test was used to compare data groups where uniformity was not confirmed. This paper contains two main types of data: survival data and in vitro blood coagulation measurement system data. Of these, the survival data was analyzed using the Kaplan–Meier method. Parallel line quantification was used to compare in vitro blood coagulation systems. Furthermore, a binary array method was used to compare blood coagulation markers. All statistical analyses described here were performed using JMP version 11 software (SAS Institute, Cary, NC, USA). In all statistical analyses, a two-tailed probability value of less than 0.05 was considered statistically significant.

## Data Availability

The original contributions presented in this study are included in the article. Further inquiries can be directed to the corresponding author.
